# The “sweet” path to cancer: focus on cellular glucose metabolism

**DOI:** 10.3389/fonc.2023.1202093

**Published:** 2023-05-25

**Authors:** Carla Iacobini, Martina Vitale, Giuseppe Pugliese, Stefano Menini

**Affiliations:** Department of Clinical and Molecular Medicine, “La Sapienza” University, Rome, Italy

**Keywords:** aerobic glycolysis, diabetes mellitus, hypoxia inducible factor (HIF)-1α, inflammation, methylglyoxal (MGO), oxidative phoshorylation, pyruvate kinase M isoform 2, Warburg effect

## Abstract

The hypoxia-inducible factor-1α (HIF-1α), a key player in the adaptive regulation of energy metabolism, and the M2 isoform of the glycolytic enzyme pyruvate kinase (PKM2), a critical regulator of glucose consumption, are the main drivers of the metabolic rewiring in cancer cells. The use of glycolysis rather than oxidative phosphorylation, even in the presence of oxygen (i.e., Warburg effect or aerobic glycolysis), is a major metabolic hallmark of cancer. Aerobic glycolysis is also important for the immune system, which is involved in both metabolic disorders development and tumorigenesis. More recently, metabolic changes resembling the Warburg effect have been described in diabetes mellitus (DM). Scientists from different disciplines are looking for ways to interfere with these cellular metabolic rearrangements and reverse the pathological processes underlying their disease of interest. As cancer is overtaking cardiovascular disease as the leading cause of excess death in DM, and biological links between DM and cancer are incompletely understood, cellular glucose metabolism may be a promising field to explore in search of connections between cardiometabolic and cancer diseases. In this mini-review, we present the state-of-the-art on the role of the Warburg effect, HIF-1α, and PKM2 in cancer, inflammation, and DM to encourage multidisciplinary research to advance fundamental understanding in biology and pathways implicated in the link between DM and cancer.

## 1 Introduction

Recent epidemiological studies have reported a transition from cardiovascular diseases to cancer as the leading cause of excess death associated with diabetes mellitus (DM) (1, 2). Cancer mortality among people with DM, especially type 2 (T2) DM, is approximately 30%-50% higher than in the general population, particularly for pancreatic, liver, colorectal, and endometrial cancers (3, 4). Clinical and preventive efforts must be directed at fighting DM-related risk factors for cancer to reduce the excess mortality risk in individuals with DM.

Possible mechanisms for a biological link between DM and cancer are hyperinsulinemia, inflammation, and hyperglycemia (5). Hyperglycemia is the distinctive feature of DM and the main cause of various life-threatening complications in both type 1 (T1) DM and T2DM (6, 7). A direct link between hyperglycemia and cancer comes from studies showing that, at high concentrations, glucose acts as DNA-damaging factor and impedes tumor suppressive functions, leading to genomic instability and eventually resulting in malignant transformation (8, 9). DM has been also associated with cancer promotion and progression (10–12); mechanisms involved in the regulation of cancer cell metabolism and the way cancer cells utilize glucose may mediate this association.

After briefly summarizing the molecular and biochemical characteristics of the Warburg effect in cancer cells, we will examine the updated evidence demonstrating similar metabolic and molecular changes in immune cells involved in inflammation and in target cells and tissues of chronic DM complications. In particular, the role of hypoxia inducible factor (HIF)-1α and M2 isoform of the glycolytic enzyme pyruvate kinase (PK) in driving the metabolic reprogramming of tumor, inflammatory, and diabetic cells will be discussed. Finally, to foster multidisciplinary investigation, the collected evidence will be illustrated in the context of a plausible hypothesis centered on changes in cellular glucose metabolism as mechanistic link between DM and cancer.

## 2 Warburg effect, HIF-1α, and PKM2 in cancer: when metabolism rhymes with opportunism

In cancer, a close relationship exists between the rate of glucose utilization and that of cell proliferation (13). In 1924, Otto Warburg identified the link between cancer and glucose by showing that tumor tissues consume and metabolize to lactate tremendous amounts of glucose relative to non-transformed tissues (14). While some cancer cells are oxidative and targeting mitochondrial oxidative phosphorylation (OXPHOS) may be a promising therapeutic target for oxidative carcinomas (15), most cancers cells exhibit suppressed mitochondrial respiration and a high rate of glucose uptake even in the presence of oxygen. This metabolic rewiring is known as both Warburg effect and aerobic glycolysis. Consistent with the importance of the Warburg effect for cancer cells, withdrawing glucose or inhibiting glycolysis is deleterious to tumorigenesis in experimental models of cancer (16, 17). How cancer cells take advantage from these metabolic changes and how glycolysis is related to cell proliferation is still not fully understood. Along with the proposal that the Warburg metabolism may be a way to produce ATP quickly (18), widely accepted hypothesis include: 1) expansion of the pool of glycolytic biosynthetic intermediates to support anabolic reactions and redox demand (19), 2) persistent NAD+ regeneration to sustain *de novo* lipogenesis (20), and 3) augmented lactate production to favor tumor growth and metastasis by affecting the tumor microenvironment (21). Along with changes in the tissue microenvironment, oncogenes and tumor suppressors that drives tumorigenesis contribute to the acquisition of the Warburg phenotype *via* activation of numerous transcription factors (including HIF-1α) regulating several genes encoding glycolytic proteins (including PKM2) (22).

HIF-1α is a master regulator of oxygen homeostasis playing a key role in the adaptive regulation of energy metabolism in mammalian tissues. By simultaneously increasing the expression of glycolytic enzymes and restraining mitochondrial function, HIF-1α can switch glucose metabolism from OXPHOS to glycolysis also in response to physiological and pathological stimuli other than hypoxia (23), including hyperglycemia-induced oxidative (24) and carbonyl (25) stress. In cancer cells, HIF-1α cooperates with the oncoprotein MYC to activate transcription of genes involved in glucose metabolism, including glucose transporters (e.g. GLUT1 and GLUT3) and glycolytic enzymes (e.g. lactate dehydrogenase A, hexokinase 2, PKM2, etc.) (26). In addition to stimulate glycolysis, HIF-1α actively represses mitochondrial respiration and biogenesis by inducing pyruvate dehydrogenase kinase 1 (27) and reducing peroxisome-proliferator-activated receptor γ co-activator-1α (28). Consistent with an important role in cancer cell biology, HIF-1α overexpression strongly correlates with poor prognosis for several solid cancers. Accordingly, pharmacological targeting of the HIF-1α signaling pathways has been recognized as a promising strategy for cancer therapy in the recent years (29).

The PKs are terminal enzymes of the glycolytic pathway that catalyze the conversion of phosphoenolpyruvate and ADP to pyruvate and ATP and are important modulators of cellular glucose metabolism. The PKM1/M2 isoforms are encoded by the same gene (*PKM*) and are generated by the alternative splicing of *PKM* mRNA (30). While PKM1 only exists as a stable and highly active tetrameric form and is expressed in most adult tissues (31), PKM2 is highly expressed during embryonic development and is reactivated in tissue regeneration and tumor development, suggesting that it is critical for actively proliferating cells (32). Unlike the constitutionally active PKM1, PKM2 is in equilibrium among the dimeric and monomer forms, which are catalytically inactive, and the active tetrameric form. Therefore, the glycolytic activity of PKM2 is subject to allosteric control (33). This implies that, at the same protein level, PKM2 is much less effective than PKM1 in catalyzing the last step within glycolysis (31). Accordingly, high ratios of PKM2/PKM1 lead to accumulation of all upstream glycolytic intermediates and diversion of metabolic flux towards the glycolytic biosynthetic branches, including the pentose-phosphate pathway, the hexosamine pathway, and the glycerol synthesis (34). This process is exploited by cancer cells to sustain their high biosynthetic and redox demand (35).

PKM2 and HIF-1α regulate each other. In fact, PKM2 is a transcriptional target of HIF-1α and a key player in the Warburg effect of glycolytic cancer cells (26). In turn, as a dimer, PKM2 translocates into the nucleus, interacts with, and promotes the transcriptional activity of HIF-1α (36). Therefore, HIF-1α and PKM2 are recognized as major drivers of cancer metabolism participating in a positive feedback loop that enhances the Warburg effect and feeds the glycolysis branch pathways (23, 30).

## 3 Warburg effect, HIF-1α, and PKM2 in inflammation: a matter of polarization

It has been almost 50 years since the first demonstration of aerobic glycolysis during lymphocyte proliferation (37). In the 2000s, some observations on metabolic reprogramming were extended to other cells of innate and adaptive immunity. Since then, a growing interest in the role of metabolism in immune regulation has bloomed. The exciting advances in the field of immunometabolism have been recently reviewed (38, 39). We summarize here the role of aerobic glycolysis, HIF-1α, and PKM2 in immune cells involved in chronic inflammation, which participates in all stages of tumorigenesis as well as DM development and progression to complications (40, 41).

In dendritic cells and macrophages, pro-inflammatory stimuli induce the shift to aerobic glycolysis and the production of inflammatory cytokines, such as interleukin (IL)-1β and tumor necrosis factor-α (TNF-α) (42). IL-1β and TNF-α are involved in insulin resistance (43, 44) and, together with other IL family members, promote tumorigenesis through complex mechanisms that involve direct growth stimulation and production of growth factors, recruitment of myeloid cells and immunosuppression, endothelial cell activation and promotion of angiogenesis (45, 46). In macrophages, the metabolic rewiring towards an enhanced glycolytic phenotype promotes polarization to the classically activated (or “M1”) phenotype and the production of many inflammatory mediators (44). Consistently, the glycolysis inhibitor 2-deoxy-D-glucose (2-DG) blocks (47), whereas GLUT1 overexpression enhances (48) M1 inflammatory functions. Conversely, OXPHOS is critical for the anti-inflammatory and tissue repair functions of alternatively activated (or “M2”) macrophages (49, 50). The balance between glycolysis and mitochondrial respiration also differentially regulates the phenotype and function of various subsets of T cells. For instance, T regulatory cells (Tregs) rely on glycolysis only during initial activation and proliferation, after that they switch toward oxidative metabolism for their regulatory functions. Consistently, GLUT1 expression increases the number of Tregs, but reduces their immunosuppressive capacity (51). Vice versa, T helper (Th)17 cells - a distinct subset of CD4+ T cells that produce the highly pro-inflammatory IL-17 – can be converted into Tregs by blocking glycolysis with 2-DG (52). Like the pro-inflammatory CD4+ Th17 cells, CD8+ lymphocytes require a Warburg-like metabolism not only for their proliferative capacity, but also for their effector functions (53, 54).

It is by now long-established that HIF-1α stabilization in immune cells can occur in an oxygen-independent manner. Bacteria and their cell membrane component lipopolysaccharide (LPS), inflammatory mediators, and endogenous molecules, such as the tricarboxylic-acid cycle intermediate succinate (47), can induce HIF-1α protein accumulation in macrophages through transcriptional and post-translational mechanisms under normoxic conditions (55–58). By cross talking with the nuclear factor-κB pathway, HIF-1α modulates essential inflammatory functions in myeloid cells (59). In keeping with a critical role for HIF-1α in the pro-inflammatory response in macrophage and T cells, HIF-1α deletion induces defective macrophage response to LPS and inhibits Th17 cell generation in mice (44, 60).

In addition to induce HIF-1α accumulation, pro-inflammatory stimuli trigger the expression of PKM2, which is now recognized as a critical determinant of the Warburg effect in macrophages. In fact, stabilizing PKM2 tetramerization with the allosteric activator TEPP-46, thus favoring PKM2 glycolytic activity and the glycolytic flux toward pyruvate, restores OXPHOS and reduces LPS-induced production of IL-1β, while promoting macrophage M2 polarization (61). In addition, PKM2 over expression induces, whereas downregulation inhibits, the activation of several toll-like receptor pathways (62). PKM2 tetramerization by TEPP-46 also blocks PKM2 nuclear translocation and restrains pro-inflammatory polarization in T cells by inhibiting the Warburg metabolism and favoring OXPHOS (63).

Overall, upregulation of aerobic glycolysis supports inflammatory immune functions. Hindering HIF-1α accumulation and PKM2 expression or favoring glycolytic activity over transcriptional activity of PKM2 by allosteric activation, blocks the Warburg metabolism and curbs inflammatory responses by supporting regulatory and anti-inflammatory immune phenotypes.

## 4 Warburg effect, HIF-1α, and PKM2 in diabetes: team members or individual runners on the road to complications?

While confirming previous findings of increased levels of glycolytic intermediates, recent omics studies in DM and related target organ damage have provided evidence of impaired mitochondrial metabolism and biogenesis, along with other features of a metabolic rewiring resembling the Warburg effect (64–66). Several metabolic intermediates and glycolytic enzymes, including PKM2, have been proposed as potential triggers of aerobic glycolysis and diversion of glycolytic intermediates into branch pathways (64).

**Figure 1:**
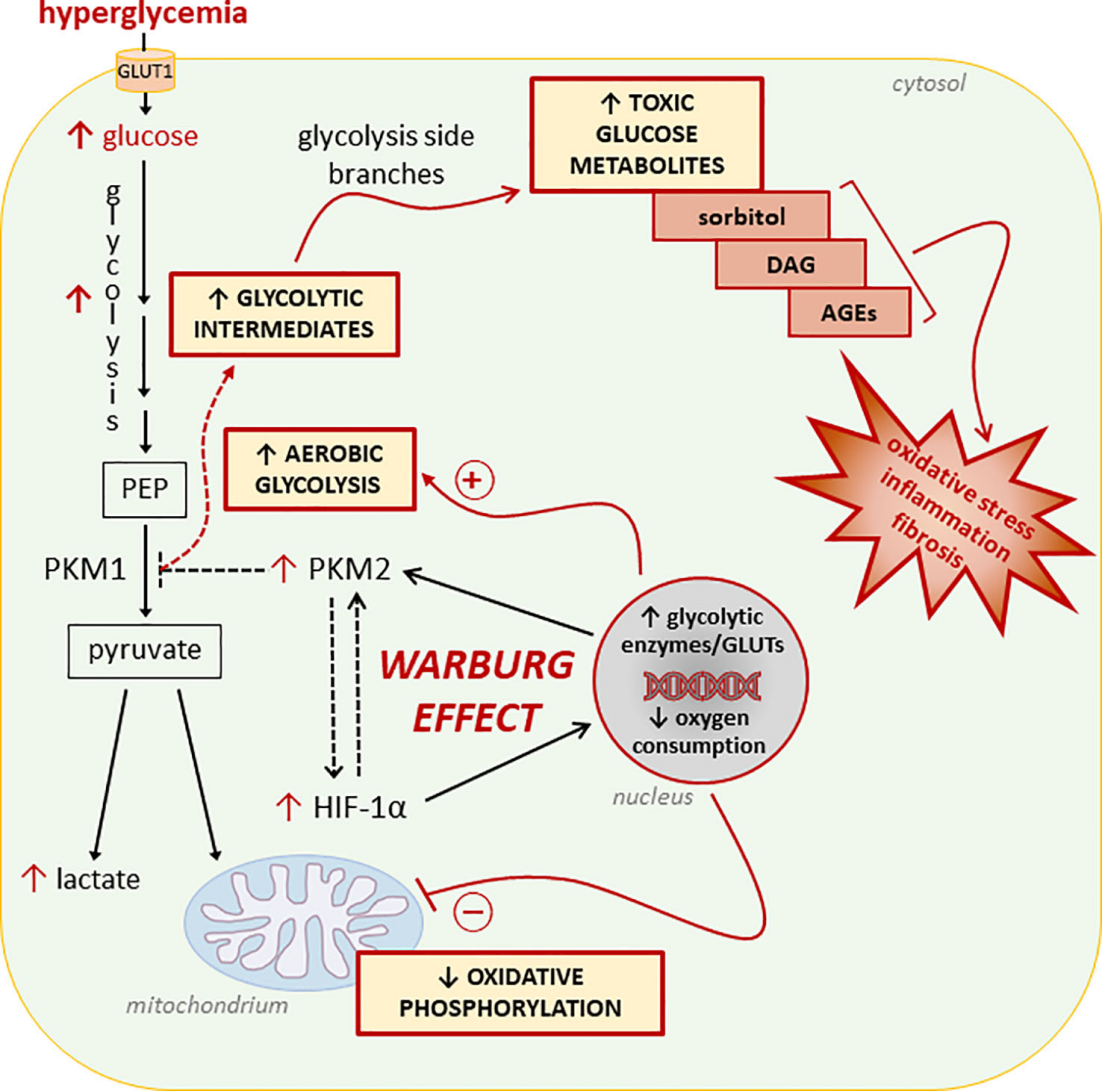
Glucose metabolic reprogramming induced by hyperglycemia in insulin-independent cells. Refer to the main text for detailed description and references. Dashed lines/arrows indicate well-established processes in cancer cells not yet confirmed in normal cells exposed to glucose concentrations in the diabetic range. In particular, the interaction between the hypoxia inducible factor 1-α (HIF-1α) and the M2 isoform of pyruvate kinase (PKM2), and the role of PKM2 in reducing the overall PK activity leading to accumulation of glycolytic intermediates need to be demonstrated. PEP, Phosphoenolpyruvate; GLUTs, glucose transporters; DAG, diacylglycerol; AGEs, advanced glycation end-products.

Diabetic complications arise in tissues that exhibit insulin-independent glucose uptake (67, 68). In the cells of these tissues, activation of aerobic glycolysis may be a consequence of increased glucose uptake from systemic circulation and an attempt to quickly metabolize excess cellular glucose (69) (**Figure 1**). The drawback of this process is the cellular accumulation of toxic glucose metabolites (66, 70). In fact, at variance with cancer cells, normal cells are not actively proliferating. Accordingly, the enhanced glucose uptake, buildup of glycolytic intermediates, and flux through the glycolytic branch pathways results in an accumulation of sorbitol, diacylglycerol, and advanced glycation end products (AGEs) leading to the activation of pro-inflammatory and -oxidative pathways (71). To our knowledge, only one study has attempted to establish a relationship between mitochondrial dysfunction, the Warburg-like metabolism, and accumulation of toxic glucose metabolites in DM. By showing that high glucose induces HIF-1α activity and a switch from oxidative metabolism to glycolysis and its principal branches, this study suggests that aerobic glycolysis may play an initiating role in glucotoxicity and diabetic complications (25).

In addition to hypoxia, numerous metabolic stressors associated with DM, including hyperglycemia, affect HIF-1α stabilization and activity. Due to the heterogeneity of the findings, what is the net impact of DM-related factors on HIF-1α signaling is a matter of debate in literature (72). Concisely, hyperglycemia seems to inhibit HIF-1α expression induced by low-oxygen conditions (73, 74), suggesting a weaker HIF-1α-dependent response to hypoxia in DM. Conversely, high glucose concentrations promote HIF-1α nuclear translocation, transcriptional activity, and lactate accumulation in normoxic conditions (25, 72, 75–78). Ultimately, these discrepancies in the effect of DM on HIF-1α signaling are likely the result of different experimental conditions (e.g., hypoxia vs normoxia) used for modulating the levels and activity of HIF-1α. In this regard, it must be remembered that the Warburg effect occurs, by definition, in the presence of normal levels of oxygen.

Recently, proteomic studies conducted in T1DM patients have demonstrated increased circulating and renal levels of mitochondrial and glycolytic enzymes in those without diabetic nephropathy (DN). Curiously, elevated levels of the underactive glycolytic enzyme PKM2 were associated with reduced renal accumulation of toxic glucose metabolites and susceptibility to DN, suggesting a protective role of PKM2 by improving cellular glucose metabolism (66, 79). However, preclinical work by the same and other investigators has shown that TEPP-46 treatment reverses metabolic abnormalities, mitochondrial dysfunction, and kidney pathology of diabetic mice by enhancing PKM2 tetramer formation (i.e., glycolytic activity) and suppressing HIF-1α and lactate accumulation (66, 80, 81). These experimental findings suggest that also in DM, as in cancer and immune cells (61, 82), PKM2 overexpression may favor glucose metabolic reprogramming towards aerobic glycolysis, accumulation of glycolytic intermediates, and pro-inflammatory signaling. Collectively, these interesting findings could lead to greater understanding of DM complications if the functional complexity of PKM2 and its role as a key regulator of glucose metabolism is considered.

In general, the body of knowledge acquired in a century of research on cancer and immune metabolism has been overlooked in the interpretation of the data concerning several aspects of cellular glucose metabolism in DM, including aerobic glycolysis, HIF-1α induction, and PKM2 expression. For example, claims that HIF-1α activity should be enhanced in DM because HIF-1α signaling is submaximal for the degree of hypoxia in diabetic tissues (83, 84) overlook the role of this transcription factor in aerobic glycolysis and inflammation. Increased cellular glucose uptake, mitochondrial dysfunction, and pro-inflammatory signaling induced by HIF-1α activity (26–28, 44) would not be at all beneficial for tissues affected by DM complications (70). Rather, regarding the role of chronic hypoxia in DM complications, it might be more biologically sound to promote the activity of the HIF-2α isoform, which has different effects to HIF-1α on glucose metabolism and even opposite effects on redox state and inflammation (72, 85). Another naive conclusion, which is inconsistent with the mechanisms that regulate cellular glucose metabolism and drive hyperglycemia-induced cell damage, is that overall PKM2 expression should be increased to enhance PK activity and prevent DM complications (80, 86). Actually, to favor glycolytic flux to pyruvate and reduce the accumulation of toxic glucose metabolites, the expression of the constitutively active enzyme PKM1 should be preferred over that of PKM2. Favor the expression of the less active isoform M2 and then have to enhance PK activity with pharmacological agents does not seem the best strategy to improve cellular glucose metabolism and prevent DM-induced target organ damage. As in cancer (87), PKM2 activators are interesting drugs to consider for complications of DM, but the simplistic view that high PKM2 levels mean high levels of PK activity must be overcome. In fact, it is the exact opposite (31, 61).

Overall, more research is needed to fully characterize the changes in cellular glucose metabolism associated with DM complications and the role of HIF-1α and PKM2 as components of a molecular network that regulates metabolic reprograming towards the Warburg effect.

## 5 Discussion

Tight glucose control is important for reduction of cancer risk in T2DM (88). Substantial evidence supports a direct causal link between DM and carcinogenesis; hyperglycemia can in fact induce malignant transformation by causing DNA damage (9), oncogenic mutations (8), and loss of tumor suppressive functions (89). Besides this, cancer and DM share similar changes in some aspects of cellular energetics, particularly glucose metabolism. Hyperglycemia leads to excessive cellular glucose uptake and changes in glucose metabolism resembling the Warburg effect, including accumulation of glycolytic intermediates (64–66). Interestingly, among the molecular mechanisms associated with the anti-tumor activity of the anti-diabetic drug metformin, suppression of the Warburg effect has also been proposed (90). The question whether the Warburg effect, besides being a consequence, might also play a causal role in carcinogenesis has been raised in the past without receiving much attention, mainly because of the lack of plausible pathomechanisms (91). However, there are numerous clues that lead us to consider the metabolic reprogramming induced by hyperglycemia as a possible field of investigation to unravel the connections between diabetes and cancer.

**Figure 2:**
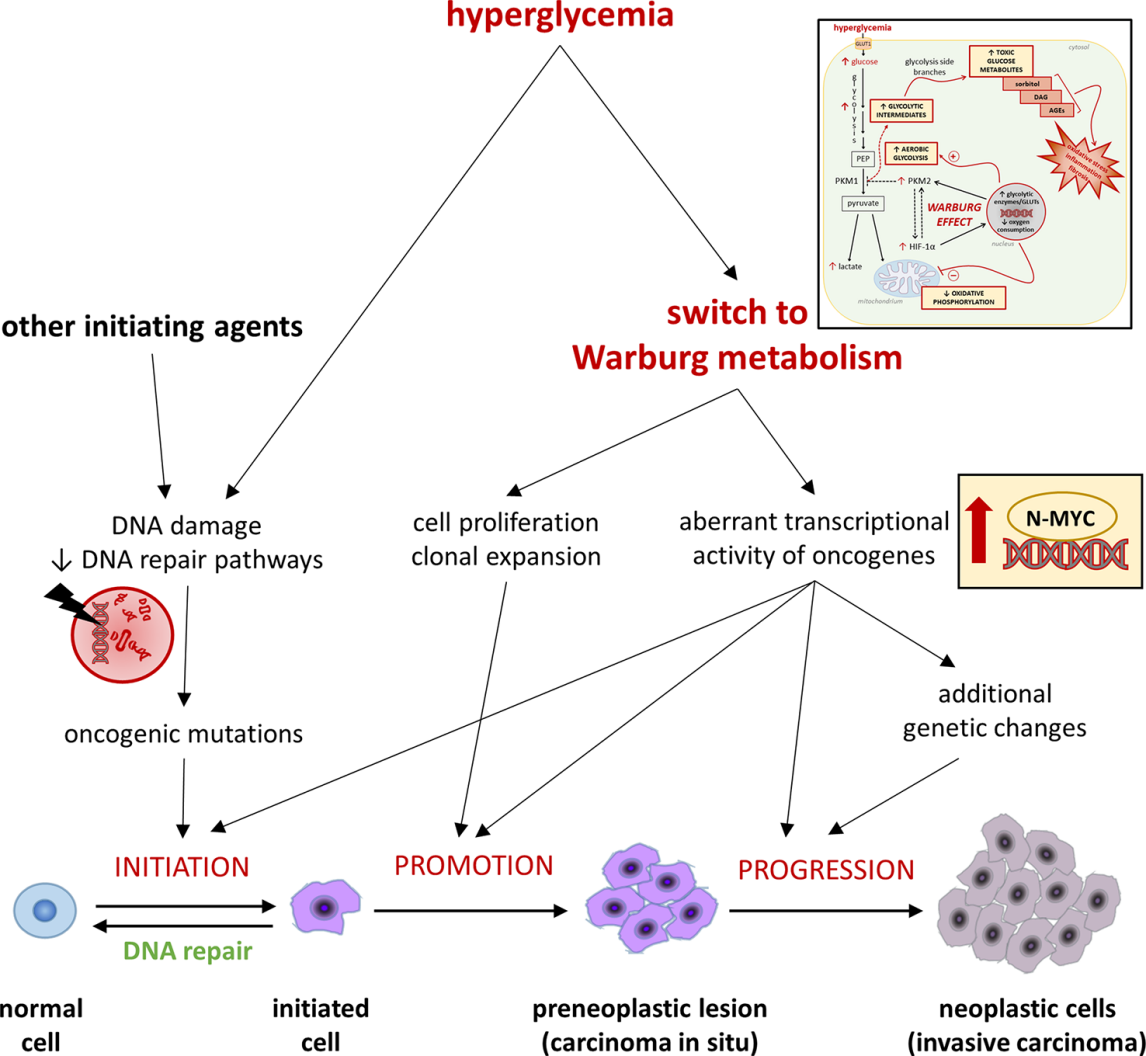
Hyperglycemia and the Warburg-like metabolism driven by excessive cellular glucose uptake in cancer initiation, promotion, and progression. Refer to the main text for detailed description and references.

Tumorigenesis comprises multiple steps of mutations subjected to a natural selection (**Figure 2**). Environmental forces and cellular adaption mechanisms that provide the mutated cell clone with survival and proliferative advantages over the neighboring cells govern this process (92). As tumor microenvironment factors influence cancer metabolism (93), hyperglycemia may promote the acquisition of a Warburg metabolism in transforming cells. In turn, hyperglycemia-mediated glycolytic reprogramming may contribute to shape the metabolic features of the evolving tumor cells by increasing the activity and fostering mutations of oncogenes regulating cell metabolism (8, 93), thus playing an active role in cancer promotion and progression. For example, high glucose was recently shown to stabilize and induce aberrant transcriptional activity of N-MYC - a member of the MYC family - even in normal cells, leading to increased proliferation and functional impairment (94). Overall, by inducing a Warburg-like metabolism, hyperglycemia might favor tumorigenesis by both contributing to the selection of more malignant phenotypes and directly inducing, in normal cells, the transcriptional activity of oncogenes that regulate multiple aspects of tumor metabolism, eventually increasing the chances of malignant transformation.

The mechanisms by which aerobic glycolysis favors cell proliferation, regulates inflammatory immune functions, and induce cell damage in DM are not yet fully elucidated. The “anabolic” (34) and “energetic” (18) hypotheses explain how the Warburg metabolism provides building blocks and an increased rate of ATP to support the anabolic and energetic demand of proliferating cells. Regardless of the discussion of their validity (18), the current hypotheses do not address the question of the causal relationship and mechanistic link between aerobic glycolysis and cell proliferation in tumor and immune cells, or cell injury in DM. Studies in the fields of immunology and metabolism have identified interesting alternative (or complementary) mechanisms that may explain how glycolytic reprogramming benefits cancer and immune cells and promotes DM complications. These mechanisms rely on the signaling function of glycolytic intermediates and/or their spontaneous decomposition products, including the inevitable side-product of glycolysis methylglyoxal (MGO). This is a highly reactive dicarbonyl compound and major precursor of advanced glycation end-products (AGEs) (95). By acting at both transcriptional and post-translational levels, MGO plays important roles in the immune response to inflammatory stimuli (96–98) and, together with AGEs, is involved in the pathogenesis of DM complications (25, 95, 99–101) and in the onset and progression of many cancers (10, 11, 102–104).

In conclusion, oncology and immunology scientists have continued to build on the seminal work by biochemists to improve understanding of glucose metabolic rewiring in cancer and immune system biology and pathology. Researchers in endocrinology and metabolism have lagged behind in this process and are struggling to put the puzzle pieces together. A multidisciplinary approach could not only help to unravel the skein and effectively interpret data for a real progress in cardiometabolic research, it also may generate new knowledge on the mechanisms linking DM and cancer.

## Author contributions

Writing—original draft preparation, CI and SM; writing—review and editing, MV and GP; visualization, SM; supervision, GP; funding acquisition, SM. All authors contributed to the article and approved the submitted version.
